# Association between severe hepatic steatosis examined by Fibroscan and the risk of high-risk colorectal neoplasia

**DOI:** 10.1371/journal.pone.0279242

**Published:** 2022-12-22

**Authors:** Kwang Woo Kim, Hyoun Woo Kang, Hosun Yoo, Yukyung Jun, Hyun Jung Lee, Jong Pil Im, Ji Won Kim, Joo Sung Kim, Seong-Joon Koh, Yong Jin Jung

**Affiliations:** 1 Department of Internal Medicine and Liver Research Institute, Seoul National University College of Medicine, Seoul, Republic of Korea; 2 Laboratory of Intestinal Mucosa and Skin Immunology, Seoul National University College of Medicine, Seoul, Republic of Korea; 3 Division of Gastroenterology, Department of Internal Medicine, Seoul National University Boramae Medical Center, Seoul National University College of Medicine, Seoul, Republic of Korea; 4 Department of Internal Medicine, Seoul National University Bundang Hospital, Seongnam-si, Republic of Korea; Osaka Rosai Hospital: Osaka Rosai Byoin, JAPAN

## Abstract

The prevalence of colorectal neoplasm in patients with non-alcoholic fatty liver disease has increased twice as high as that in the general population. FibroScan is a new modality for evaluating hepatic steatosis. This study aimed to investigate the relationship between the risk of high-risk colorectal neoplasia and hepatic steatosis examined using FibroScan. This was a cross sectional study of prospectively enrolled subjects who were scheduled to undergo index colonoscopy as a health screening between March 2018 and February 2019. The severity of steatosis was graded as normal, mild, moderate, or severe using FibroScan. A total of 140 consecutive subjects were enrolled and sequentially examined using FibroScan and colonoscopy. Subjects with hepatic steatosis had more high-risk colorectal neoplasia than those without hepatic steatosis. In addition, tumor size was larger in subjects with hepatic steatosis. In multivariable analysis, severe hepatic steatosis was an independent risk factor for high-risk colorectal neoplasia (adjusted odds ratio: 3.309, confidence interval: 1.043–10.498, *p* = 0.042). Alcohol consumption was also identified as a risk factor for high-risk colorectal neoplasia. In conclusion, severe hepatic steatosis on FibroScan is associated with the development of high-risk colorectal neoplasia. Thus, physicians should be aware of the association between colorectal neoplasia and hepatic steatosis assessed by FibroScan and its clinical implications.

## Introduction

Colorectal cancer is the third most prevalent cancer and the second most common cause of cancer-related mortality worldwide, respectively [1]. The adenoma-carcinoma sequence plays a key role in colon carcinogenesis [2]. The process takes about 10 years, presenting an opportunity to decrease the incidence and mortality by detecting and removing the adenomatous lesion during this period. Screening tests and therapeutic interventions for colorectal adenoma are crucial for reducing the mortality of colorectal cancer [3–5]. In addition, early detection with screening tests such as fecal occult blood test, flexible sigmoidoscopy, or colonoscopy has been shown to reduce the incidence and mortality of colorectal cancer [6–10]. Therefore, the current guidelines for colorectal cancer screening recommend regular checkups in adults [11,12]. However, colonoscopy carries the risk of serious complications such as perforation, requires rigorous bowel preparation, and is expensive. Therefore, information regarding high-risk patients with advanced colorectal neoplasia is crucial to select the most appropriate modalities for colorectal cancer screening.

Non-alcoholic fatty liver disease (NAFLD) is a risk factor for colorectal cancer and adenoma [13–17]. Previous epidemiologic studies have demonstrated that the prevalence of NAFLD was about 25% worldwide and 30.3% in Korea [18,19]. NAFLD includes simple hepatic steatosis and non-alcoholic steatohepatitis. Colorectal adenoma is 1.5 times higher in patients with NAFLD than in the general population [13,16]. This means that the importance of NAFLD in colorectal tumorigenesis is increasing. A previously suggested mechanism of the relationship between NAFLD and colorectal tumorigenesis is that the pro-inflammatory status, presence of metabolic syndrome, increased insulin resistance, and decreased adiponectin in patients with NAFLD may be associated with an increased risk of colorectal adenoma [20]. NAFLD represents a chronic systemic inflammatory state characterized by increased inflammation. This chronic inflammatory condition or secretory molecules such as reactive oxygen species, interleukin (IL)-6, and tumor necrosis factor (TNF)-α could be associated with the occurrence of colorectal adenoma [13]. However, the precise mechanism underlying the association between NAFLD and colorectal tumorigenesis remains unclear.

Liver biopsy is the gold standard for evaluating hepatic steatosis and fibrosis. However, it is invasive and can cause complications, such as massive bleeding. Abdominal ultrasonography is the standard modality for evaluating NAFLD; however, it is a subjective procedure and thus has limitations due to inter-observer variation [21]. FibroScan is a novel modality for evaluating fatty liver disease and has the advantages of convenience, reproducibility, and non-invasive [22]. It measures a novel, non-invasive value called the controlled attenuation parameter (CAP) by acquiring a considerable part of the liver volume. The accuracy of CAP in the evaluation of hepatic steatosis has been examined in many studies, including the studies of NAFLD [23]. However, no prospective studies on the relationship between hepatic steatosis proven by FibroScan and colorectal neoplasia have been conducted to date.

Thus, this study aimed to evaluate the relationship between the risk of colorectal tumorigenesis examined by index colonoscopy and hepatic steatosis examined by FibroScan.

## Materials and methods

### Study population

This was a cross sectional study of prospectively enrolled subjects who were scheduled to undergo index colonoscopy as a health screening between March 2018 and February 2019 at Seoul National University Boramae Hospital, Seoul. Consecutive subjects aged 40 to 75 years who underwent a healthcare screening exam not related with symptom of colitis, bleeding or drug history at Seoul National University Boramae Hospital were screened. The exclusion criteria were (1) previous colonoscopy, colon study, or computed tomographic colonography; (2) history of colorectal cancer or inflammatory bowel disease such as ulcerative colitis and Crohn’s disease; (3) previous colectomy for any cause; or (4) history of liver disease (e.g., chronic hepatitis due to hepatitis B or C virus), liver cirrhosis of any cause, alcoholic liver disease, or autoimmune hepatitis.

The study protocol was approved by the Institutional Review Board of Seoul National University Boramae Hospital (Protocol number: 30-2018-18) and was conducted according to the tenets of the Declaration of Helsinki. Written informed consent was obtained from all enrolled patients.

### Data collection

Detailed history taking, electronic medical record search, and laboratory examinations were performed at the first visit. History taking included age; sex; body mass index (BMI); alcohol consumption; smoking; underlying diseases such as hypertension, diabetes, liver disease, and gastrointestinal disease; and history of surgery, colonoscopy, or radiologic examination. Laboratory examination included complete blood count, chemistry, and serological tests such as hepatitis B and C viruses.

BMI was calculated as weight (kg) divided by height (m) squared (kg/m^2^). BMI ≥ 23 kg/m^2^ was classified as overweight or obese according to the Asia-Pacific BMI criteria established by the Western Pacific Region of the World Health Organization [24]. Current alcohol consumption was defined as current alcohol drinking or within 1 year. Subjects who drank more than 10 g of alcohol per day were excluded from the study. Current smokers were defined as those who smoked currently or within 1 month.

Fibroscan was performed within 4 weeks from the first visit and proceeded before the colonoscopy on the same day.

### Colonoscopy

Colonoscopy was performed by a board-certified gastroenterologist (S.K., expert with more than 10,000 colonoscopy procedures performed) using CF- and CF-H colonoscopies (Olympus, Tokyo, Japan). The procedure was performed under conscious sedation with intravenous midazolam and intramuscular pethidine. The subjects were given polyethylene glycol for bowel preparation with detailed instructions.

A complete colonoscopic examination was defined as reaching the medial side of the ileocecal valve, as documented by endoscopic imaging. The withdrawl time of colonoscopic evaluation was at least 6 minutes to minimize the probability of missing lesions. Incomplete colonoscopic examinations were excluded from the analysis. Images of all examined colorectal lesions were collected to determine the total number of lesions, locations, and sizes. The quality of bowel preparation was graded using the Boston Bowel Preparation Scale [25]. Procedures graded as “poor,” defined as a subscore of 0 or 1 in any of the segments during colonoscopy, were excluded from analysis. All abnormal mucosal lesions were biopsied or resected using endoscopic mucosal resection, if possible. Lesion size was assessed using the width of the biopsy forceps or measured after resection or surgery.

Colorectal adenomas were confirmed by pathologic evaluation after colonoscopic tissue biopsy or mucosal resection. A diagnosis of total colorectal neoplasia was made if the lesion was confirmed as adenoma or cancer, irrespective of other findings such as size, number, dysplasia, and detailed histology. High-risk colorectal neoplasia was defined as advanced colorectal neoplasia or ≥ 3 colorectal adenomas. Advanced neoplasia was defined as advanced adenoma or colorectal cancer. Advanced adenoma was defined as the presence of one or more of the following features: (1) largest diameter ≥ 1 cm, (2) including tubulovillous or villous histology confirmed by pathologic examination, and (3) high-grade dysplasia confirmed by pathologic exam [26]. Serrated lesions that measured at least 1 cm, included dysplasia, or were confirmed as traditional serrated adenomas were classified as high-risk colorectal neoplasia. In cases of multiple lesions, the most advanced pathology was selected as the definitive lesion. Non-advanced adenoma was defined as an adenoma measuring < 10 mm in size with low-grade dysplastic changes and < 25% villous component. Nonspecific inflammation or hyperplastic lesions, such as hyperplastic polyps, were classified as normal.

### FibroScan measurement

FibroScan was used evaluate hepatic steatosis and fibrosis, which were graded by severity [27]. The examination was performed by trained physicians or nurses who were certified by the manufacturer and blinded to the endoscopic and histological examinations. The model used was FibroScan 502 with both M and XL probes. The probes were chosen automatically by the software in the FibroScan according to the distance from the skin to the liver capsule. It was performed on the right side of the body, with the probe located on the skin of the intercostal area at the level of the liver with the right angle. The patient was placed in a supine position with the right arm raised. The examiner located the probe away from the hepatic vasculature and measured the thickness and volume of the target at least 6 cm in thickness and 3 cm^3^, respectively.

The quantitative values of steatosis and fibrosis were measured according to CAP and liver stiffness measurement (LSM), respectively. FibroScan measures LSM and CAP based on vibration-controlled transient elastography (VCTE). For LSM, VCTE uses pulse-echo ultrasonic acquisition to quantify the speed of shear waves induced in liver tissue; for CAP, ultrasonic attenuation of the echo wave is acquired [28,29]. The results of CAP and LSM are expressed as dB/m and kPa, respectively. The results of the test were accepted as an average value after 10 or more valid individual measurements. The severity of steatosis was graded as normal, mild, moderate, and severe according to the CAP score defined by the cut-off value of FibroScan; these were marked as normal, S1, S2, and S3, respectively. The values were measured according to the manufacturer’s guidelines. The cut-off values of the CAP score were ≥ 238 dB/m, ≥ 260 dB/m and ≥ 290 dB/m for S1, S2 and S3, respectably. Under the value of S1 grade was described as normal. The grade of fibrosis was defined by the cut-off values of LSM by Fibroscan: the values were ≥ 7.5 kPa, ≥ 10 kPA and ≥ 14 kPa for F2, F3 and F4, respectively. The value of < 7.5 kPa was described as F0-1.

### Outcomes

The primary outcome measure was the correlation with high-risk colorectal neoplasia according to the severity of hepatic steatosis because high-risk colorectal neoplasia requires surveillance colonoscopy in 3–5 years, which is earlier than the usual schedule [26]. Additionally, the predictability for the presence of colorectal adenoma according to the severity of hepatic steatosis was also analyzed.

### Statistical analysis

Continuous variables were expressed as the means with standard deviations or as percentages, as appropriate. Categorical variables were expressed as numbers or as percentages, as appropriate.

Categorical variables were compared using one-way ANOVA or χ^2^ test, as appropriate. Univariable and multivariable logistic regression analysis were performed to assess the interaction between the dependent variables of colorectal adenomas and the independent variables including test results of hepatic steatosis, age, sex, BMI, smoking, alcohol consumption, underlying diseases, family history of colorectal cancer, and related laboratory results. Each odds ratio (OR) is presented together with its 95% confidence interval (CI). All statistical analyses were performed using the Statistical Package for Social Sciences version 25.0 (SPSS Inc., Chicago, IL, USA). Statistical significance was defined as a two-sided *p* value of < 0.05.

## Results

### Baseline demographic and clinical characteristics

Of the 155 consecutive subjects who were enrolled before the endoscopic procedure, 15 patients were excluded because they did not undergo FibroScan or colonoscopy. Finally, a total of 140 consecutive subjects who underwent index colonoscopy for screening were enrolled and evaluated using FibroScan. The mean subject age was 59.2 years (range, 40–75 years), and there were 63 (45%) and 77 (55%) female and male subjects, respectively. There were 49 subjects (35%) who were current alcohol drinkers.

The population was initially categorized as having steatosis, as measured by FibroScan. Subjects with S1 or higher steatosis were categorized into the steatosis group, while subjects with normal values were categorized into the normal group. There was no significant between-group difference in age, sex, height, smoking, underlying diseases, family history of colorectal cancer, and laboratory test results. However, body weight and BMI were significantly different between the two groups. HDL levels were significantly lower in the steatosis group. ALT and TG levels were significantly higher in the steatosis group. The baseline subject characteristics by steatosis grade are shown in Table 1.

**Table 1:** Baseline characteristics of the study population.

Characteristic	Normal (n = 66)	Hepatic steatosis (n = 74)	*p*-value
**Median age (range, year)**	60 (39–74)	60 (39–75)	0.729
**Male gender (%)**	35 (53.0)	42 (56.8)	0.661
**Height (cm)**	163.7 **±**8.7	164.3 **±**9.0	0.694
**Body weight (kg)**	62.7 ±10.1	70.4 ±12.6	<0.001
**BMI (kg/m** ^**2**^ **)**	23.3 ±2.6	25.9 ±3.1	<0.001
**Smoking, current (%)**	10 (15.2)	19 (25.7)	0.123
**Alcohol consumption, current (%)**	24 (36.4)	25 (33.8)	0.752
**Underlying illness**			
** Hypertension (%)**	19 (28.8)	25 (33.8)	0.528
** Diabetes (%)**	6 (9.1)	8 (10.8)	0.737
**Family history of colorectal cancer (%)**	4 (6.1)	8 (10.8)	0.313
**Median CAP score (range, dB/m)**	213.5 (101–235)	282.5 (238–372)	<0.001
**Median LSM score (range, kPa)**	3.7 (2.4–75)	4.1 (2.3–11.4)	0.442
**Laboratory results (mean±SD)**			
** Total bilirubin (mg/dL)**	0.9 ±0.4	0.8 ±0.3	0.174
** ALP (IU/L)**	66.0 ±18.9	71.6 ±19.0	0.085
** AST (IU/L)**	25.2 ±9.9	27.1 ±11.8	0.322
** ALT (IU/L)**	21.0 ±10.4	29.9 ±19.2	0.001
** γGT (IU/L)**	32.5 ±60.0	30.1 ±27.5	0.757
** Total cholesterol (mg/dL)**	181.5 ±38.8	176.1 ±46.3	0.313
** Triglyceride (mg/dL)**	109.5 ±58.1	151.6 ±92.7	0.002
** HDL (mg/dL)**	58.8 ±18.4	46.7 ±10.9	<0.001
** LDL (mg/dL)**	101.8 ±30.3	107.7 ±31.7	0.270

SD, standard deviation; BMI, body mass index; ALP, alkaline phosphatase; AST, aspartate aminotransferase; ALT, alanine aminotransferase; γGT, gamma-glutamyl transpeptidase; HDL, high density lipoprotein; LDL, low density lipoprotein.

### Hepatic steatosis and fibrosis

All data on hepatic steatosis and fibrosis were assessed using FibroScan and are summarized in Table 2. There were 74 (53%) and 66 (47%) subjects with hepatic steatosis and normal liver, respectively. In the hepatic steatosis group, 21 (28%), 19 (26%), and 34 (46%) subjects had S1, S2, and S3 hepatic steatosis, respectively. Overall, 135 (96%) of the study subjects had normal or mild hepatic fibrosis, and only 5 (4%) subjects had a hepatic fibrosis of grade 2 or higher.

**Table 2:** Hepatic steatosis and fibrosis of the study population.

Characteristic	Normal (n = 66)	Hepatic steatosis (n = 74)	*p*-value
**Hepatic steatosis grade (%)**			NA
** Normal**	66 (100)	NA	
** S1**	NA	21 (28.4)	
** S2**	NA	19 (25.7)	
** S3**	NA	34 (45.9)	
**Hepatic fibrosis grade (%)**			0.755
** F0-1**	64 (97.0)	71 (95.9)	
** F2**	1 (1.5)	2 (3.0)	
** F3**	0	1 (1.4)	
** F4**	1 (1.5)	0	

NA, not applicable; F0-1, no or mild hepatic fibrosis.

### Colorectal neoplasia according to the presence or absence of hepatic steatosis

In total, 74 (53%) subjects had one or more adenomas. There was no significant difference in the rate of total colorectal neoplasia between the two groups. However, there was a significant difference in high-risk colorectal neoplasia and non-advanced adenoma. In addition, subjects with hepatic steatosis had larger adenomas (0.6±0.5 vs. 0.4±0.5, *p* = 0.016). The rate of high-risk colorectal neoplasia was also higher in subjects with severe hepatic steatosis (44%) than in subjects with normal (20%) or mild to moderate hepatic steatosis (30%). Overall, 3 (2.1%) subjects were diagnosed with colorectal cancer. The colonoscopic findings are summarized in Table 3.

**Table 3:** Colonoscopy findings according to the presence of hepatic steatosis on FibroScan.

Endoscopic findings	Normal (n = 66)	Hepatic steatosis (n = 74)	*p*-value
**Total colorectal neoplasia† (%)**	35 (53.0)	39 (52.7)	0.969
**Non-advanced adenoma‡ (%)**	21 (31.8)	12 (16.2)	0.030
**High-risk colorectal neoplasia§ (%)**	13 (20)	27 (36)	0.028
**Advanced neoplasia**‖ **(%)**	11 (16.7)	20 (27.0)	0.141
**Colorectal cancer (%)**	2 (3.0)	1 (1.4)	0.493
**Advanced adenoma¶ (%)**	9 (13.6)	19 (25.7)	0.075
** High-grade dysplasia**	0 (0)	0 (0)	NS
** ≥1 cm sized adenoma**	9 (13.6)	19 (25.7)	0.075
** Villous adenoma**	2 (3.0)	1 (1.4)	0.484
** Serrated adenoma**	1 (1.5)	2 (2.7)	0.639
**Three or more adenomas (%)**	3(4.5)	11(14.9)	0.059
**Average number of adenomas (mean±SD)**	0.9 ±1.3	1.4 ±1.9	0.097
**Average size of adenomas (cm, mean±SD)**	0.4±0.5	0.6±0.5	0.016

SD, standard deviation; NS, not significant.

† Total colorectal neoplasia was confirmed as adenoma or cancer, irrespective of other findings such as size, number, dysplasia and detailed histology.

‡ Non-advanced adenoma is defined as an adenoma < 10 mm in size with low-grade dysplastic changes and < 25% villous component.

§ High-risk colorectal neoplasia was defined as advanced colorectal neoplasia or 3 or more colorectal adenomas. Serrated lesion of which size was 1 cm or over, included dysplasia, or was confirmed as traditional serrated adenoma was classified as high risk colorectal neoplasia.

‖ Advanced neoplasia was determined as advanced adenoma or colorectal cancer.

¶ Advanced adenoma was defined if the lesion including serrated adenoma has one or more features of followings; (1) largest diameter ≥ 1 cm, (2) including tubulovillous or villous histology confirmed by pathologic exam, (3) including high grade dysplasia confirmed by pathologic exam.

### Risk factors of high-risk colorectal neoplasia

In the univariable analysis, BMI, smoking, and severe hepatic steatosis were to be significant influencing factors of high-risk colorectal neoplasia. In the multivariable analysis, severe hepatic stenosis and alcohol consumption were found to be significant factors for high-risk colorectal neoplasia (adjusted OR: 3.309, 95% CI: 1.043–10.498, *p* = 0.042; adjusted OR: 2.876, 95% CI: 1.049–7.883, *p* = 0.040, respectively) (Table 4).

**Table 4:** Risk Factors for the presence of high risk colorectal neoplasia.

Factors	Univariable analysis		Multivariable analysis	
	OR (95% CI)	p-value	OR (95% CI)	p-value
Age†	1.244 (0.455–3.404)	0.671	2.549 (0.707–9.191)	0.153
**Male Gender**	1.330 (0.632–2.801)	0.453	0.815 (0.308–2.152)	0.679
BMI‡	3.187 (1.222–8.318)	0.018	2.634 (0.920–7.537)	0.071
Smoking§	3.051 (1.303–7.143)	0.010	1.755 (0.567–5.435)	0.329
Alcohol consumption§	2.111 (0.994–4.486)	0.052	2.876 (1.049–7.883)	0.040
**Hypertension**	1.714 (0.795–3.697)	0.169	1.570 (0.602–4.097)	0.357
**Diabetes**	1.444 (0.453–4.611)	0.535	0.809 (0.198–3.313)	0.769
**Family history of colorectal cancer**	0.474 (0.099–2.265)	0.349	0.315 (0.055–1.805)	0.195
**Hepatic steatosis grade**				
**S1**	2.038 (0.684–6.071)	0.201	2.667 (0.788–9.029)	0.115
**S2**	1.456 (0.444–4.775)	0.535	1.259 (0.315–5.039)	0.745
**S3**	3.219 (1.297–7.988)	0.012	3.309 (1.043–10.498)	0.042
Hepatic fibrosis grade¶	1.702 (0.274–10.588)	0.569		
**Laboratory results**				
**Total cholesterol**	0.998 (0.989–1.006)	0.615	1.001 (0.991–1.011)	0.822
**Total bilirubin**	0.304 (0.085–1.083)	0.066		
**ALP**	1.000 (0.980–1.019)	0.968	0.997 (0.973–1.022)	0.820
**AST**	0.988 (0.950–1.026)	0.524	0.977 (0.905–1.054)	0.542
**ALT**	1.002 (0.980–1.025)	0.864	0.995 (0.951–1.041)	0.832
**γGT**	1.000 (0.993–1.008)	0.903		
**Triglyceride**	1.004 (0.999–1.008)	0.112		
**HDL**	0.990 (0.966–1.014)	0.402		
**LDL**	1.005 (0.993–1.017)	0.427		

OR, odds ratio; CI, confidence interval; BMI, body mass index; ALP, alkaline phosphatase; AST, aspartate aminotransferase; ALT, alanine aminotransferase; γGT, gamma-glutamyl transpeptidase; HDL, high density lipoprotein; LDL, low density lipoprotein.

† 50 year-old or above.

‡ 23 kg/m^2^ or above.

§ current state.

¶ grade 2 or above.

## Discussion

Hepatic steatosis and colorectal neoplasia may be associated by sharing common factors such as insulin resistance by metabolic syndrome and altered gut microbiome [30–32]. However, the association between hepatic steatosis and colorectal tumorigenesis remains unclear because most studies had a retrospective design and because of the heterogeneous colonoscopy examination. In addition, no prospective study has evaluated the association between hepatic steatosis assessed using FibroScan and colorectal neoplasia. This study found that severe hepatic steatosis on FibroScan is associated with an increased risk of high-risk colorectal neoplasia in the asymptomatic general population.

Colon tumorigenesis comprises three processes: initiation, promotion, and progression. The role of hepatic steatosis in colon tumorigenesis remains unclear. However, previous studies have demonstrated that fat accumulation in hepatocytes can lead to an inflammatory state in the liver. Hepatic steatosis can then induce or accelerate the disruption of the gastrointestinal tract via pro-inflammatory cytokines (e.g., TNF-α, IL-6, and IL-8) that promote tumor formation in the colon [13]. It also induces insulin resistance, resulting in an increase in insulin-like growth factor (IGF), which can lead to gut inflammation and colorectal carcinogenesis [33,34].

In our study, the prevalence of total colorectal neoplasia did not differ significantly according to the presence of hepatic steatosis, suggesting that hepatic steatosis is not associated with tumor initiation in colorectal tumorigenesis. However, there were significant differences in high-risk colorectal neoplasia and non-advanced adenoma between the steatosis and normal groups. Moreover, the average tumor size was greater in the steatosis group, suggesting that hepatic steatosis may be associated with tumor promotion in colorectal tumorigenesis. Further studies are needed to elucidate the basic mechanism of the effect of hepatic steatosis on colon tumorigenesis and the therapeutic effect of hepatic steatosis on colorectal tumor promotion.

We demonstrated that alcohol consumption was an independent risk factor for high-risk colorectal neoplasia. This is in line with a previous report by Terry et al. that alcohol consumption could be a risk factor for advanced colorectal adenoma [35]. Song et al. also showed that alcohol intake could increase the risk of both advanced adenoma and more than three adenomas [36]. In our study, subjects with alcoholic hepatitis were excluded. In addition, there was no difference in alcohol consumption according to the presence or absence of hepatic steatosis, suggesting that alcohol consumption itself has a direct effect on colorectal tumorigenesis. Therefore, patients with hepatic steatosis need to abstain from drinking to reduce the risk of colorectal neoplasia. Further studies are needed to elucidate the mechanism of the synergistic effect between alcohol consumption and hepatic steatosis in colon tumorigenesis.

In general, colorectal cancer screening is recommended for subjects aged ≥ 50 years. However, colonoscopy as the reference standard for detecting precancerous lesions and colorectal cancer is an intensive procedure, requiring bowel preparation and having risks of various complications such as abdominal discomfort, pain, and perforation. In addition, colonoscopy is expensive, which may cause a delay in colorectal cancer screening. Therefore, it is crucial to know the risk factors for high-risk colorectal neoplasia. FibroScan is an easy procedure with high reproducibility. It also has the advantages of providing more information than does liver biopsy by acquiring images of a larger liver volume and being non-invasive, requiring only a probe to touch the skin. Severe hepatic steatosis is prevalent in 24.2% of the general population. In this study, approximately half of the subjects with severe hepatic steatosis had high-risk colorectal neoplasia. This indicates that physicians should recommend the individuals with severe hepatic steatosis for colonoscopy for colorectal cancer screening rather than other alternative methods.

Our study has several strengths. To our best knowledge, this is the first study to explore the impact of hepatic steatosis assessed using FibroScan on the risk of colorectal neoplasia in the general screening population who underwent their first colonoscopy during lifetime. Our results provide further evidence on the role of severe hepatic steatosis in colon tumorigenesis, and our protocol overcomes the bias in previous studies. Second, all subjects were studied per standardized study protocol and only subjects with NAFLD were included, while those with hepatitis-related confounding factors such as viral infection or autoimmune disease were excluded. This allowed us to explore the independent role of hepatic steatosis in colon tumorigenesis. Third, we evaluated confounding factors including smoking, BMI, family history of colorectal cancer, and underlying diabetes. Finally, all polyps identified during the study were removed and histologically evaluated by pathologists. Therefore, our study provides a robust causal relationship between severe hepatic steatosis and high-risk colorectal neoplasia.

However, our study had some limitations. First, this was a single-nation, single-race, single-center study, and thus, the results may have limited generalizability. Second, the study population was not large and we could not evaluate confounding factors such as abdominal visceral adiposity and show more clear statistical values in various aspects of colorectal neoplasia. Third, there was few data about advanced liver fibrosis, the relationship between the liver fibrosis measured by Fibroscan and colorectal neoplasia could not be statistically evaluated. In addition, although hepatic steatosis examined by FibroScan was well correlated with the result of liver biopsy, the possibility of selection bias is unavoidable because the prevalence of moderate to severe hepatic steatosis in this study is higher than that in the general population [19,37–39]. Finally, we choose only one of the reference values that have been previously reported. There are various reference values of CAP and LSM for assessing hepatic steatosis and fibrosis from previous studies. It means that the cut-off values could vary from many factors such as countries, ethnics, statistical methods, underlying clinical characteristics and so on [40–42]. Further large-scale, prospective studies are required to confirm our results and the effect of managing hepatic steatosis in reducing colorectal tumorigenesis.

In conclusion, severe hepatic steatosis on FibroScan is significantly associated with high-risk colorectal neoplasia by index colonoscopy. Therefore, physicians and endoscopists should be aware of the association between colorectal neoplasia and hepatic steatosis assessed using FibroScan and its clinical implications.

## Supporting information

S1 Data (XLSX)
